# Etiologic Pattern, Severity, and Outcome of Pericardial Effusion Among Children Seen Over Ten Years at a Tertiary Hospital in Sokoto, Northwest Nigeria

**DOI:** 10.7759/cureus.74485

**Published:** 2024-11-26

**Authors:** Khadijat O Isezuo, Usman M Sani, Usman M Waziri, Bilikisu I Garba, Abubakar Umar, Solomon I Ukwuani, Moyijo Maishanu, Inalegwu C Udah, Sirajo Shehu, Muideen A Ajadi, Yahaya Mohammed

**Affiliations:** 1 Department of Pediatrics, Usmanu Danfodiyo University Teaching Hospital, Sokoto, NGA; 2 Department of Surgery, Usmanu Danfodiyo University Teaching Hospital, Sokoto, NGA; 3 Department of Medical Microbiology, Usmanu Danfodiyo University Teaching Hospital, Sokoto, NGA

**Keywords:** children, pericardial effusion, severity, sokoto, spectrum

## Abstract

Introduction: Pericardial effusion (PE) is an abnormal accumulation of fluid in the pericardial space, which, if severe, is associated with high mortality. The causes are diverse, including infective and non-infective. Few studies have looked at the spectrum of severity and causes in Northern Nigeria. The aim was to determine the spectrum of causes and severity of pericardial effusion, as well as the outcome among children seen at the Paediatric Cardiology Unit, Usmanu Danfodiyo University Teaching Hospital (UDUTH), Sokoto, Nigeria.

Methods: This ten-year retrospective study reviewed the echocardiographic and admission records of children admitted to the Pediatric Cardiology unit, UDUTH, Sokoto, from January 2014 to December 2023. Data on age, gender presentation, diagnosed causes, and outcomes were extracted and entered into a study pro forma.

Results: Cases with pericardial effusion were 121. Of these, 79 (65.3%) were male and 42 (34.7%) were female (M:F = 1.9:1). The mean age of all cases was 8.2±4.3 years, and 72% (87/121) were aged 5 years or older. Eighteen (14.8%) had severe effusion and cardiac tamponade, 42 (34.7%) had moderate effusion, and 61 (50.4%) had mild effusion. Infective causes were 91 (75.2%) and included rheumatic heart disease (30/121, 24.8%), tuberculosis (28/121, 23.1%), and dilated cardiomyopathy (15/121, 12.4%), while non-infective causes were 30 (24.8%) and included congenital heart disease (14/121, 11.6%), pulmonary hypertension (7/121, 5.8%), and connective tissue diseases (4/121, 3.3%). Five had open tube pericardiostomy, and 10 had percutaneous echo-guided drainage. Two cases with sepsis were positive for microbial growth, and two cases of tuberculous effusion had chronic inflammation on pericardial biopsy. Outcomes differed by underlying cause, with mortalities mainly from rheumatic heart disease and dilated cardiomyopathy.

Conclusion: In the study area, preventable infective causes of pericardial effusion predominated with higher mortality. Males and older children had more severe effusions. More efforts at prevention would be beneficial in this regard.

## Introduction

Pericardial effusion (PE) is an increase in the physiologic amount of fluid within the pericardial space. This usually results from inflammation of the pericardium [1]. The causes are diverse and include infective and non-infective etiologies. In developing countries, tuberculosis and purulent effusions caused by bacterial agents are the primary causes, while viral infections and postsurgical complications are the most frequent causes in developed countries [2]. Other causes include inflammatory, autoimmune, uremic, and malignant etiologies [2]. The amount of pericardial fluid accumulation ranges from mild to severe. Mild fluid collections are insignificant or result in minor pathophysiologic consequences. When the amount of fluid in the non-distensible pericardial space becomes excessive, pressure within the pericardium increases and is transmitted to the heart [1].

Diagnosis of PE may be difficult if only clinical examination findings are used. Only significant pericardial effusion may present with signs and symptoms that include tachycardia, raised jugular venous pressure, orthopnea, a drop in blood pressure of at least 10% during inspiration (pulsus paradoxus), and pericardial rub [2]. Some of these symptoms could also overlap with those of the preceding causes, such as lung parenchymal infections, heart failure, and inflammatory causes; hence, if not diagnosed and treated early, it could result in severe cardiovascular compromise leading to death. This phenomenon called cardiac tamponade results from impaired filling of the heart due to the pericardial fluid compressing the chambers [1, 2]. The effect on the cardiac chambers and resulting clinical symptoms also depends on the rapidity of fluid accumulation, such that small volumes of effusions can cause cardiac tamponade when they occur rapidly, while chronic and slow PE allows the elastic pericardium to stretch, thereby causing minimal effects on the hemodynamics till a significant volume has accumulated [3].

Echocardiography is the imaging modality of choice to assess the pericardium [2]. Pericardial effusion is graded by echocardiography into mild, moderate, and severe when PE measured is <10 mm, 10-20 mm, and >20 mm, respectively [4]. Many studies have reported mainly on the causes and outcomes of significant PE, which are usually dramatic in presentation and warrant immediate intervention with management challenges, especially in resource-constrained countries like Nigeria [5].

This study retrospectively assessed by echocardiography the spectrum of severity of PE, associated causes, and outcomes among children managed at the Pediatric Cardiology unit, Usmanu Danfodiyo University Teaching Hospital (UDUTH), Sokoto, Nigeria.

## Materials and methods

The study was conducted at the Pediatric Cardiology Unit of UDUTH, Sokoto. The hospital is a major tertiary referral center for pediatric cardiac cases in the North-Western region of Nigeria and the neighboring countries of Niger and Benin. The hospital also has other major specialties and sub-specialties, including the cardiothoracic surgical unit, with whom some of the patients were co-managed.

Data were collected from echocardiographic reports and admission records for children who had an echocardiographic diagnosis of pericardial effusion and who were seen and followed up in the Pediatric Cardiac Unit or Cardiothoracic Surgical Unit over the 10-year period from 1st January 2014 to 31st December 2023.

Relevant information was extracted and entered into a study pro forma sheet, which included age, gender, year of diagnosis, underlying diagnosis with pericardial effusion, severity of effusion, and outcome. Excluded were those that had suggestive symptoms and chest X-ray features but no echocardiographic confirmation.

This research was approved by the ethical review committee of UDUTH, Sokoto, as part of an echocardiographic review with approval number UDUTH/HREC/2021/1024/V1. Consent-taking was not applicable as it was a retrospective review with no sensitive data. Occlusion of identifiers from images was done to maintain the confidentiality of data. Data entry was also coded with numbers, not name identifiers, and applications containing data had passwords with limited access.

Echocardiography was done using 2-D and Doppler transthoracic echocardiography (TTE) in the supine and left lateral positions via the standard parasternal, apical, and subcostal views using the Hewlett Packard Sonos 5500 echocardiography machine (Bloomfield CT 06002, USA) and a 5 & 8 MHz transducer. The echocardiographic diagnostic criteria used was an echo-free space between the visceral and parietal pericardium of < 10 mm = mild effusion, 10-20 mm = moderate effusion, and > 20 mm = severe effusion [4]. Collapse of the right atrium in systole and right ventricle in diastole was diagnosed as tamponade. While dilation of the inferior vena cava (IVC), fibrinous deposits, organized collection, and pericardial thickness were all noted [4].

The specific diagnosis, either congenital or acquired heart disease, was confirmed by 2 or 3 cardiologists. Other extra-cardiac diagnoses were made by the specialties involved in their care. This was done in addition to other cardiac assessments, including history, physical examination, chest X-rays, and electrocardiography. Other investigations, such as complete blood counts, acute phase reactants, sputum microscopy, culture, and electrolyte levels, were also done as necessary.

Those with significant effusion and tamponade had percutaneous echo-guided drainage or open tube pericardiostomy for those with thick purulent collections and posteriorly located collections. Aspirates were sent for microscopy culture and sensitivity, acid-fast bacilli test, GeneXpert (Cepheid, California, USA), and biochemistry were done within 1 to 6 weeks as indicated to monitor patients who presented for follow-up. Those who had surgical intervention also had pericardial biopsy and histology done.

Data were analyzed using IBM Corp. Released 2017. IBM SPSS Statistics for Windows, Version 25.0. Armonk, NY: IBM Corp. Independent variables included age at diagnosis, year of diagnosis, gender, and cause of PE, and the severity of PE (mild, moderate, severe) were dependent variables. Univariate analysis (mean, median, standard deviation) was done for continuous variables, such as age, while frequency tables, charts, and proportions were used for the categorical variables, such as gender, diagnosis, and year of diagnosis. Bivariate analysis (chi-square or Fisher’s exact test) was used to assess the relationship of age category and gender to the severity and causes of PE. Mean ages of the different degrees of severity of PE were compared with ANOVA. The level of statistical significance was set at p-value < 0.05.

## Results

Out of the 2147 children who had echocardiography during the 10-year period, 121 (5.6%) had differing degrees of pericardial effusion (Table 1). The mean age of all patients with effusion was 8.2±4.3 years, and the majority of patients, 87/121 (72%), were aged 5 years and older (χ2=4.2, p=0.04). There were 79 (65.3%) male patients and 42 (34.7%) were female, with a ratio of 1.9:1 (Table 1).

Infectious causes were responsible for 91 (75.2%) of all cases. Rheumatic heart disease was the topmost cause, accounting for 30 (24.8%), followed closely by TB, which accounted for 28 (23.1%), and myocarditis/dilated cardiomyopathy with 22 (18.2%). Non-infectious causes were 28 (23.1%) and topped by CHD with 14 (11.6%) cases, respectively. Details of the aetiologies are seen in Table 1. Some had multiple diagnoses, as seen in Table 1 footnotes. Three cases of tuberculosis had viral co-infection.

**Table 1:** Characteristics of subjects with pericardial effusion according to age, gender, causes, and severity The data has been represented as N, %; Chi-square analysis was used; p-value was considered significant at < 0.05. ∞ one case had TB/HIV co-infection, another had active hepatitis B virus infection, and another had herpes zoster. * Pulmonary HTN Causes: sickle cell cardiomyopathy, adenoidal hypertrophy, post-pertussis, idiopathic. $ Connective tissue diseases: Systemic Juvenile Idiopathic Arthritis, Systemic Lupus Erythematosus. # Others: diagnosis not established.

Patient Characteristic (n=121)	All patients	Male	Female	Test of significance
Age				
≤ 5 years	34 (28.1%)	27 (22.3%)	7 (5.8%)	χ^2^ = 4.2 p = 0.04
> 5 years	87 (71.9%)	52 (43.0%)	35 (28.9%)	
Diagnosis				
Infectious cause (n=91)				
Acute rheumatic fever/Rheumatic heart disease	30 (24.8%)	15 (12.4%)	15 (12.4%)	
Tuberculosis	28 (23.1%)	19 (15.7%)	9 (7.4%)	
Myocarditis/Dilated cardiomyopathy	22 (18.2%)	14 (11.6%)	8 (6.6%)	
Sepsis	8 (6.6%)	6 (5.0%)	2 (1.6%)	
Diphtheritic myocarditis	3 (2.5%)	0 (0.0%)	3 (2.5%)	
Non-infectious cause (n=28)				
Congenital heart disease	14 (11.6%)	12 (9.9%)	2 (1.7%)	
Pulmonary hypertension*	7 (5.8%)	6 (5.0%)	1 (0.8%)	
Connective tissue diseases^$^	4 (3.3%)	4 (3.3%)	0 (0.0%)	
Endomyocardial fibrosis	2 (1.7%)	2 (1.7%)	0 (0.0%)	
Uraemia/Chronic kidney disease	1 (0.83%)	0 (0.0%)	1 (0.83%)	
No diagnosis established (n=2)				
Others	2 (1.7%)	1 (0.83%)	1 (0.83%)	
Severity of pulmonary effusion				
Mild	61 (50.4%)	35 (28.9%)	26 (21.5%)	χ^2^ = 2.4 p = 0.32
Moderate	42 (34.7%)	31 (25.6%)	11(9.1%)	
Severe	18 (14.8%)	13 (10.7%)	5 (4.1%)	

Apart from equal gender occurrence of PE in RHD/ARF and female preponderance of diphtheritic myocarditis, all other diagnoses were predominant among the males. Details are seen in Table 1. More than half of the cases, 61 (50.4%), had a mild degree of effusion, while the least proportion, 18 (14.8%), had severe effusion. The mean age of those with mild effusion was 7.1±4.4 years, while for moderate effusion it was 8.9±4.1, and for severe effusion was 10.5±2.8 (F=4.9, p=0.009). 

**Figure 1:**
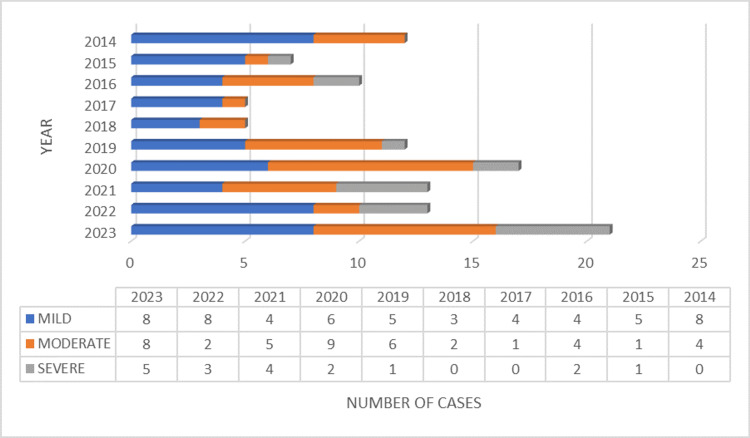
Trend in number of cases and severity of pericardial effusion over 10 years

Figure 1 below shows the yearly incidence of pericardial effusion. The trend shows that the number of patients with pericardial effusions increased from the year 2020 to 2023 with a rising number of cases of severe effusions.

**Figure 2:**
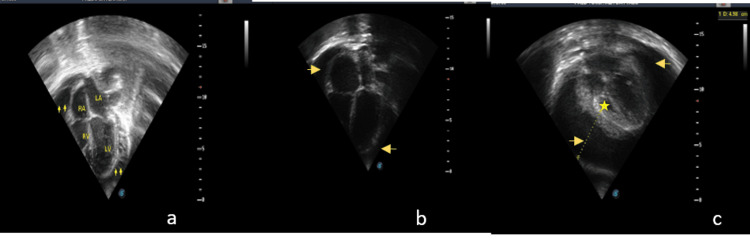
Echocardiography of three patients showing (a) apical 4-chamber view with mild circumferential effusion with fibrin strands (arrows), (b) apical 4-chamber view with moderate clear effusion (arrows), (c) sub-xiphoid view with severe effusion and fibrin deposits, thickened pericardium (arrows), and collapse of right ventricle (star)

Tuberculosis (11/18; 61.1%) and sepsis (4/18; 22.2%) were the main causes of severe effusion, while moderate effusion was still topped by TB (14/42; 33.3%), followed by RHD (12/42; 28.6%). Mild effusion was mainly caused by myocarditis/DCM (19/61; 31.1%), followed by RHD (17/61; 27.9%). These are shown in Table 2. Figure 2 shows three echo images (a, b, c) with different severity of PE ranging from mild, moderate, to severe.

**Table 2:** Distribution of severity of pericardial effusion according to cause The data has been presented as N, %

Diagnoses	Mild (N=61)	Moderate (N=42)	Severe (N=18)	Total (N=121)
Rheumatic heart disease and Acute rheumatic fever	17 (27.9)	12 (28.6)	1 (5.6)	30 (24.8)
Tuberculosis^∞^	3 (4.9)	14 (33.3)	11 (61.1)	28 (23.1)
Myocarditis/dilated cardiomyopathy	19 (31.1)	3 (7.1)	0 (0.0)	22 (18.2)
Congenital heart disease	8 (13.1)	6 (14.3)	0 (0.0)	14 (11.6)
Sepsis	3 (4.9)	1 (2.4)	4 (22.2)	8 (6.6)
Pulmonary hypertension	5 (8.2)	1 (2.4)	1 (5.6)	7 (5.8)
Connective tissue diseases	3 (4.9)	1 (2.4)	0 (0.0)	4 (3.3)
Diphtheric myocarditis	2 (3.3)	1 (2.4)	0 (0.0)	3 (2.5)
Endomyocardial fibrosis	0 (0.0)	1 (2.4)	1 (5.6)	2 (1.7)
No cause established	1 (1.6)	1 (2.4)	0 (0.0)	2 (1.7)
Uraemia/Chronic kidney disease	0 (0.0)	1 (2.4)	0 (0.0)	1 (0.83)
Total	61 (50.4)	42 (34.7)	18 (14.8)	121 (100.0)

Among those with severe effusion, fever and difficulty in breathing (13/18, 73.3%) and cough (12/18, 66.7%) were the most common symptoms (Table 3). Body swelling and chest pain were the least common (both: 10/18, 55.6%). Diffuse apex beats and muffled heart sounds were predominant amongst the signs (both: 15/18, 83.3%). Blood pressure was taken in only five (27.8%) patients and was not recordable due to cardiac tamponade.

**Table 3:** Documented presenting features of severe effusion (n=18) The data is presented as N, %

Clinical Features	N	%
History		
Fever	13	73.3
Difficulty In Breathing	13	73.3
Cough	12	66.7
Weight Loss	11	61.1
Chest Pain	10	55.6
Body Swelling	10	55.6
Examination		
Diffuse Apex Beat	15	83.3
Distant Heart Sounds	15	83.3
Small Volume Pulse	11	61.1
Raised JVP	8	44.4
Unrecordable Blood Pressure	5	27.8

Fifteen of the 18 patients (83.3%) with severe effusion had procedural drainage. Eleven (61.1%) had per-cutaneous echo-guided drainage (PCD), while four patients (22.2%) had open tube drainage (OTD). Therefore, 15 patients out of 121 had procedural drainage, as shown in Table 4. Other treatments received depended on the cause.

**Table 4:** Shows the details of the treatment received and type of drainage procedure done OTD: open tube drainage; PCD: per-cutaneous drainage Data presented as N, %

Diagnoses	Number of cases	Main treatment Received	Procedure done
Rheumatic heart disease and Acute rheumatic fever	30	Anti-failure regimen & anti-inflammatory drugs	-
Tuberculosis	28	Anti-tuberculous therapy	OTD (3), PCD (8)
Myocarditis/dilated cardiomyopathy	22	Anti-failure regimen	-
Congenital heart disease	14	Anti-failure regimen	-
Sepsis	8	Antibiotics	PCD (3)
Pulmonary hypertension	7	Pulmonary anti-hypertensive agents	-
Connective tissue diseases	4	Immunosuppressive therapy	-
Diphtheric myocarditis	3	Anti-failure regimen, antibiotics, Anti-toxin	-
Endomyocardial fibrosis	2	Anti-failure regimen	OTD (1)
No cause established	2	Steroids, diuretics	-
Uraemia/Chronic kidney disease	1	Haemodialysis	-
Total	121 (100)		15 (12.4)

Forty-six (38%) out of the total patients with effusion died, while 43 (35.5%) were lost to follow-up, and 32 (26.4%) were still on follow-up at the time the data was compiled (Table 5). The details of the outcome of each diagnosis are also depicted in Table 5.

**Table 5:** Showing the outcome among different causes of pericardial effusion Data is presented as N, %

Diagnoses	Number of cases	Outcome		
		On follow-up	Lost to follow-up	Died
Rheumatic heart disease and acute rheumatic fever	30	9 (7.4)	12 (9.9)	9 (7.4)
Tuberculosis^∞^	28	9 (7.4)	14 (11.6)	5 (4.1)
Myocarditis/dilated cardiomyopathy	22	3 (2.5)	8 (6.6)	11 (9.1)
Congenital heart disease	14	4 (3.3)	5 (4.1)	5 (4.1)
Sepsis	8	5 (4.1)	0 (0.0)	3 (2.5)
Pulmonary hypertension	7	2 (1.7)	1 (0.83)	4 (3.3)
Connective tissue diseases	4	0 (0.0)	2 (1.7)	2 (1.7)
Diphtheric myocarditis	3	0 (0.0)	0 (0.0)	3 (2.5)
Endomyocardial fibrosis	2	0 (0.0)	0 (0.0)	2 (1.7)
No cause established	2	0 (0.0)	1 (0.83)	1 (0.83)
Uraemia/Chronic kidney disease	1	0 (0.0)	0 (0.0)	1 (0.83)
Total	121	32 (26.4)	43 (35.5)	46 (38.0)

The majority of those with acute rheumatic fever/rheumatic heart disease had mild effusion and were treated with an anti-failure regimen and anti-inflammatory medications for those with acute/recurrent rheumatic fever. However, 12 (9.9%) were lost to follow-up, while 9 (7.4%) died during the course of the illness (Table 5).

Nineteen (86.3%) of 22 cases of DCM had mild effusions, with 3 (13.6%) cases having moderate effusion. They were managed with diuretics, ACE inhibitors, and inotropes. Eleven (9.1%) died, which accounted for the highest mortality.

Supportive evidence of tuberculosis in these patients included prolonged length of illness with fever, cough, weight loss, and night sweats. Examination findings revealed they were all chronically ill with significant respiratory distress, reduced pulse volume, cardiomegaly, and muffled heart sounds. All had elevated white cell count and erythrocyte sedimentation rate. Three had positive Mantoux tests. Three had viral co-infection, namely retroviral infection, active hepatitis B viral infection, and herpes zoster infection. All were prescribed anti-tuberculous therapy.

**Figure 3:**
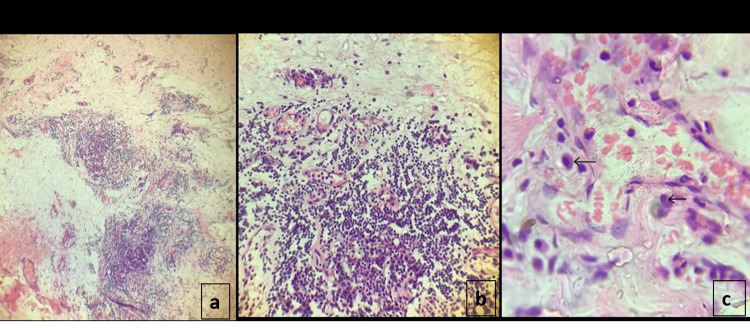
(a) H&E x 100 showing fibrous tissue with multiple foci of inflammatory infiltrates, (b) H&E x 400 showing mononuclear cell infiltrates composed of lymphocytes and plasma cells, (c) H&E x 1000 (with oil immersion) shows plasma cells (black arrows) composed of round eccentric nuclei with perinuclear halos

Out of the total cases, mortality from TB ranked third alongside CHD with five cases (4.1%). However, TB also accounted for a high rate of loss to follow-up. Out of the 28 cases of tuberculosis, 11 (39.3%) had severe effusion. Three (10.7%) had open tube pericardiostomy, while eight (28.6%) had percutaneous echo-guided drainage. Purulent effluent was seen in seven (25.0%) of the cases, while two (7.1%) had serosanguinous effluent, and one (3.6%) had hemorrhagic effusion. All were negative for acid-fast bacilli. Pericardial biopsy revealed evidence of chronic inflammation in two (7.1%) cases, including the child with hemorrhagic effusion who had recurrent collection and subsequently had sub-total pericardiectomy. Two patients died after an emergency open pericardiostomy. Figure 3 below shows the pericardial tissue histology slides stained with hematoxylin & eosin (H&E) from a patient showing chronic inflammation with plasma cells and lymphocytes.

Three of the eight (37.5%) patients with sepsis had per-cutaneous drainage, which showed purulent effusion, and microbial growth was positive in 2 (25.0%) cases (*Staphylococcus aureus* and *Streptococcus pneumoniae*). They responded to antibiotics and were discharged.

Of the 14 cases with CHD, three (21.4%) had ventricular septal defect, three (21.4%) had patent arterial duct (PDA), three (21.4%) had tetralogy of Fallot (TOF), and three (21.4%) had Ebstein anomaly; one (7.1%) had Eisenmenger syndrome, and another had a supra-mitral membrane. The majority had mild effusions; however, five (35.7%) cases had moderate effusions. The five cases with moderate effusion were two (14.3%) cases of TOF (one post total correction), two (14.3%) cases of Ebstein anomaly, and the case of Eisenmenger syndrome. Those with mild effusions responded to diuretics, while the complex cases (Ebstein & Eisenmenger) died subsequently. Tetralogy of Fallot cases were lost to follow-up. One of the PDAs closed spontaneously.

The two cases with endomyocardial fibrosis had moderate and severe effusion, respectively. The latter presented in tamponade and had open drainage twice (recurrence). He subsequently succumbed to the illness.

Two (50%) out of four cases with autoimmune disorders were confirmed to be systemic onset juvenile idiopathic arthritis, and another had systemic lupus erythematosus. Both succumbed to the illness despite immunosuppressive therapy.

The cases with pulmonary hypertension included a case of sickle cell cardiomyopathy and two cases of adenoidal hypertrophy (one had pertussis). Others were idiopathic pulmonary hypertension.

## Discussion

This study reviewed and analyzed the causes of PE diagnosed by echocardiography over a ten-year period. The causes were mainly of infective origin, especially severe effusions.

There were 121 children with varying degrees of PE during the period of 10 years, and this represented 5.6% of children who had echocardiography during the period, which was close to 4.2% reported years ago from Jos, Nigeria, but higher than 0.8% of the children seen in a seven-year period in a study in Tanzania by Zuechner [6]. This study focused on the spectrum of pericardial effusion among children seen at echocardiography, unlike Zuechner [6], who looked at the pattern of heart diseases primarily in children who presented for echocardiography. An earlier study in the center reported a lower figure of 0.3% of total patients who had echocardiography being diagnosed with pericardial effusion, but it did not assess the full spectrum of severity, as mild and moderate cases were not captured independently of their other cardiac diagnosis [7]. Most other reports studied the proportion of those with PE and compared it to the number of children admitted, thereby having different denominators [8-11].

The mean age of those with PE was 8.2 years, and more than 70% were aged above five years. Likewise, the mean age of the patients increased with the severity of the PE. The results also showed that most of the causes were more in those above five years except for CHD. This suggests that the causes of PE were more common in older children. Supporting this finding were mean ages reported from other studies of 4.8 years by Agawaral [12], 5.8 years by Sadoh [13], 6.4 years by Rima [14], and 8.1 years by Bagri [11]. Kumar [15] also reported a mean age of 8.3 years in those with tuberculous pericarditis. Sadoh [13] showed equal incidence of pericardial disease in those less than and above five years, but no further details on the individual causes were given.

Males were more affected, with a male-to-female ratio of 1.9 to 1. Similar ratios were reported in Bagri [11] and Mehdizadegan’s [10] study, with even higher male preponderance in Agrawal’s [12] study from India and Igoche’s [8] from northwest Nigeria. More males seem to be affected by the infective causes than females based on their exposure to causative agents and relatively lower immunity from their single X chromosome [16]. This was similarly reported by Okeniyi [5], from Ilesa, South Nigeria. Males predominated in all causes of PE in this study, which include infective and non-infective causes. There was equal gender prevalence for RHD, and only females with diphtheria had PE. Some studies have reported a higher incidence of diphtheria in females [17], and cardiac complications from diphtheria were higher in females in a study reported by Garud [18]. It was also observed in the literature that most studies on the cardiac complications of diphtheria dwelt on cardiac dysfunction and arrhythmias with no report on pericardial effusions.

The yearly trend showed that the proportion of those with PE was higher in recent years as compared to previously. Quite a number of cases with severe effusion were seen in the year 2023. Some authors have reported that the increase in rates of acute pericarditis with effusion in recent years could be related to a post-COVID syndrome or long COVID syndrome. Though most of these reports were from the adult population, some were seen in children [19]. Chacko also reported pericardial effusion to be a delayed complication of COVID-19 infection [20]. Though the children in this study were not tested for COVID-19, which could have been an added differential diagnosis.

The most common cause of PE was ARF/RHD, which was closely followed by TB and then DCM. These acquired heart diseases cause fluid retention and inflammation as part of their pathogenesis, thereby resulting in PE. Heart failure of various origins was the most common cause of PE in Sadoh’s [13] study in Benin City, Nigeria, while TB predominated in other studies like Igoche’s [8] study in Kano, Northwest Nigeria, as well as Bagri’s [11], Rima’s [14], and Kumar’s [15] studies in Bangladesh and India, respectively. Sepsis was the fifth most common cause in this series, whereas it topped Okeniyi’s [5] study in Nigeria and Agrawal’s [12] study in India. Pyogenic effusion was closely followed by tubercular effusion in the study by Malgope in India [21].

Only one case of CKD with uremia causing PE was seen in this study, while it was the second most common in Igoche’s [8] study in Kano and in Mehdizadegan’s [10] study in Iran; renal failure was the most common. This brings to the fore that infective and non-infective causes can contribute significantly to PE depending on location and disease patterns. Mehdizadegan [10] also opined that the predominant cases presenting to their facility had renal problems because there was a standard dialysis center located there.

Congenital heart diseases also contributed to the causes of PE in this study, with mainly milder forms of effusion. There was a patient who had PE weeks after TOF total corrective surgery. Generally, post-surgical cases of CHD have a risk of PE, especially with right-sided lesions and with the use of cardiopulmonary bypass [22]. Another patient with Eisenmenger syndrome had moderate effusion. Pericardial effusion can occur in Eisenmenger syndrome, and usually, it is a manifestation of right heart failure [23]. Right-sided HF is also the likely cause of the moderate PE seen in two of our patients with Ebstein anomaly, a CHD known for its varied presentations [24].

Regarding the severity of effusions, mild effusion accounted for 50.4% of the cases, while severe effusion was 34.7% and severe 14.8%. Tuberculosis was the predominant cause of moderate and severe effusions. This was somewhat similar to Malgope’s [21] study, where 43.3% of patients had mild, 36.7% had moderate, and 20% had severe PE. Pyogenic effusion was predominant in their study. In the study by Amare in Ethiopia [9], mild, moderate, and severe pericardial effusion was documented in 22.5%, 46.5%, and 31% of the study subjects, respectively. However, the individual causes according to the severity were not analyzed.

Mehdizadegan [10] reported minimal to mild effusion predominated in 62.4%, while moderate was 17.3% and severe cases were 15.3%. The etiological spectrum showed moderate to severe effusions were mainly due to renal causes, then post-cardiac surgery before sepsis. Tuberculosis was much less common, accounting for two cases, possibly reflecting the advancement in prevention in that area. Tuberculosis as a cause of severe effusion was also reported in other studies [8,11,13].

It is noteworthy that the diagnosis of TB was made in this study based on the clinical history, examination findings, and some supportive investigations (leucocytosis, elevated ESR, positive Mantoux test) because most of the pericardial fluid drained for those who had interventions was clinically sterile even when purulent. The study by Amare in Ethiopia [9] revealed a lower prevalence of TB than ours and captured those without growth as clinically sterile PE. Only 4.2% (3 of 71) were diagnosed with TB in their study. This was also supported by the shorter duration of symptoms of their subjects, unlike in this study where the duration of symptoms was longer with other suggestive symptoms. Generally, the yield in diagnoses of TB from pericardial fluid is low. It can be as low as 30%, and pericardial biopsy & histology may be more useful as it showed a higher yield of up to 90% [25]. However, histological yield was still low in this study, as chronic inflammation was seen in 2 cases out of 5 who had pericardial biopsy.

Viral co-infection may also be a factor in pericardial infection, as three had confirmed viral infections of HIV, hepatitis, and herpes zoster infection, respectively. These viruses have been documented as causes of pericardial effusion on their own [26]. In a South African study, Mayosi [27] reported up to half of patients with large effusions were HIV positive, warranting testing for the infection in those that present with PE.

It has been reported that symptoms and signs may not be significant in the presence of even a severe effusion, and a high index of suspicion is required [1,5,14]. Fever was the most common presenting symptom, while chest pain and body swelling were the least similar to what was reported by Rima [14]. Diffuse apex beats and distant heart sounds were the commonest symptoms. Respiratory distress was the most common in Amare’s [9] study, while cough was less common. The clinical presentations of our series were also similar to Malgope’s [21], from India, where fever, cough, and tachypnea were predominant symptoms; however, muffled heart sounds were seen in only 50% of the cases.

Pertaining to the treatment procedure, 12.4% of the total in this study required pericardial drainage, similar to 14.1% by Mehdizadegan [10]. However, in the report by Amare [9], 52.1% had pericardial drainage done, probably because they had a higher proportion of moderate and severe effusions compared to our study. Of the 18 with severe effusions in this study, 83.3% had procedural drainage as compared to 92.3% in the study by Rima [14]. These findings probably reflect the increasing availability of expertise in tertiary facilities for emergency drainage of severe effusions. Amare [9] reported that pericardial drainage was inversely proportional to mortality among their patients.

Mortality was high in this study compared to other reports at 38% compared to Amare's [9] study of 12.7%. However, most of the mortality in this report was due to the disease processes causing mild to moderate effusions like DCM and RHD. Tuberculosis was the 3rd most common cause of mortality, despite causing the bulk of severe effusions, probably due to the available life-saving procedural interventions and anti-tuberculous therapy.

This study is limited by being a retrospective review; some cases may have been missed. Also, some of those with mild effusions were lost to follow-up, and possible progression and resolution were not documented.

## Conclusions

More cases of pericardial effusion were in males and above five years. Rheumatic heart disease with mainly mild to moderate PE was the commonest cause of effusion, while TB caused especially moderate to severe effusion requiring drainage. Mortality was high with the acquired heart diseases. Many patients have been lost to follow-up.

Ensuring BCG vaccination, which prevents severe forms of TB, is essential. Other preventable programs like RHD primordial, primary, and secondary prevention should be improved. Poverty alleviation, social support, and health insurance to assess early diagnosis and treatment are essential. Enhancing follow-up with calls, home, and community visits should be encouraged.
